# ADvAnced PhysioTherapy in MuSculosKeletal Triage: Investigating prognostic factors, healthcare utilisation and clinical outcomes (ADAPT MSK) - a cohort study protocol.

**DOI:** 10.12688/hrbopenres.13769.1

**Published:** 2023-12-06

**Authors:** Fiona Callan, Louise Keating, Sarah Casserley-Feeney, Helen P. French

**Affiliations:** 1 School of Physiotherapy, Royal College of Surgeons in Ireland, Dublin, Ireland; 2 National Musculoskeletal Triage Initiative, National Clinical Programme for Trauma & Orthopaedic Surgery (NCPTOS), Royal College of Surgeons in Ireland, Dublin, Ireland

**Keywords:** Musculoskeletal triage; physiotherapy; orthopaedic triage, rheumatology triage; predictors of outcome; musculoskeletal pain, healthcare utilisation, cohort study

## Abstract

Background: Clinical specialist physiotherapist-led musculoskeletal triage clinics were introduced nationally in Ireland in 2011 to improve patient care and reduce waiting times for secondary care orthopaedics and rheumatology. Evidence has shown them to be effective in reducing waiting lists, however there are currently no data on longitudinal patient outcomes following clinic attendance. The primary aim of this cohort study is to identify predictors of pain and function outcomes up to one year following musculoskeletal triage review. Secondary aims include measuring self-reported use of healthcare resources over the 12-month follow-up period and to explore musculoskeletal phenotypes based on established prognostic factors for musculoskeletal pain. This is a prospective cohort study.

Methods: ADvAnced PhysioTherapy in MuSculosKeletal Triage (ADAPT MSK) will recruit a cohort of 252 adults through musculoskeletal triage clinics across five secondary care sites in Ireland. The STrengthening the Reporting of Observational studies in Epidemiology (STROBE) guidelines will be adhered to for future reporting. Adults (≥ 18 years old) attending physiotherapist-led musculoskeletal triage clinics with musculoskeletal pain, who do not require surgical or consultant-led medical care will be considered for participation. Participant demographics, health literacy, healthcare utilisation, and self-report questionnaires on pain, function, musculoskeletal health, musculoskeletal risk stratification, fear of movement, and psychological distress will be obtained at baseline, with follow-ups at three, six, and 12 months. Primary outcomes are pain intensity and function. Secondary outcomes include musculoskeletal risk stratification status, musculoskeletal health, healthcare utilisation, and work-related factors. Descriptive statistics will be used to profile the participants and predictors of outcome will be assessed using multivariable linear regression. Musculoskeletal phenotypes will be explored using latent class analysis.

Results: Results will be disseminated via peer-reviewed journal publication and presentation at national and international conferences. Engagement with a public patient involvement (PPI) panel will explore dissemination strategies for public and service user engagement.

## Introduction

Musculoskeletal (MSK) pain, which includes conditions such as low back pain, neck pain or osteoarthritis is recognised as one of the leading causes of disability worldwide ^1^, resulting in increased healthcare expenditure and longer waiting times for orthopaedic and rheumatology outpatient services ^2, 3^. Adult orthopaedic services represent the largest waiting list in Ireland (June 2023) with a total of 64,867. Up to 25% of patients are waiting more than 12 months for orthopaedic (22%) and rheumatology (25%) appointments in secondary care ^4^.

In 2011, to reduce outpatient Orthopaedic and Rheumatology waiting times in Ireland, the Health Service Executive (HSE) National Clinical Programmes for Trauma and Orthopaedics (NCPTOS), and Rheumatology (NCPR) established the National MSK Triage Initiative, consisting of 24 clinical specialist physiotherapist (CSP) posts in 18 Acute Hospital sites nationwide. In these MSK Triage clinics, CSPs triage patients on outpatient orthopaedic and rheumatology waiting lists, who are unlikely to require consultant care, onto appropriate care pathways. In a national audit, over 80% of patients presenting to MSK-triage clinics in Ireland were managed independently by the CSP, with 71% discharged at their initial appointment ^5^ and 23% referred to physiotherapy ^6^. From 2012 to 2018, 125,852 patients on orthopaedic and rheumatology waiting lists were managed through MSK triage services ^7^. Access to primary care physiotherapy also presents a barrier to patients, with 56,200 on primary care waiting lists and 22% (12,502) waiting greater than one year to access primary care physiotherapy services in 2022 ^8^. Longer waiting times to access physiotherapy can negatively affect patients’ quality of life, psychological wellbeing, healthcare utilisation, health outcomes and economics ^3, 9, 10^.

Whilst the National MSK Triage Initiative has been successful in reducing acute hospital outpatient orthopaedic and rheumatology waiting lists, the high discharge rate of 71% at initial appointment ^5^ warrants further examination to explore the patient journey and potential reasons why patients are not referred to the right service at the right time, in line with the Irish government health reform plan (Sláintecare) ^11^. It is possible that suboptimal access to primary care services, may be influencing referrer behaviour and decision making.

Several predictors of pain and functional outcomes in MSK conditions have previously been identified, including baseline function, pain intensity, mental well-being, co-morbidities, age, body mass index (BMI), duration of symptoms, workers’ sick leave, education level ^12^ and altered pain processing ^13^; which can also predict non-response to physiotherapy ^14^. Recently, MSK core outcome sets, and prognostic stratification tools (such as the Subgroups for Targeted Treatment Back (STarT Back) and Subgroups for Targeted Treatment MSK (STarT MSK)), have been developed, based on established prognostic factors ^12, 15^, and validated to identify earlier, those at risk of developing persistent MSK pain ^16, 17^. Research to date has shown that MSK triage is an effective waiting list initiative with good service user and healthcare professional satisfaction ^5, 7, 18– 21^. However, currently, patient outcomes, prognostic stratification, and predictors of outcome up to 1-year later have not been consistently studied in patients attending MSK triage clinics in Ireland or internationally.

### Objectives

The primary aim of this prospective, cohort study is to identify predictors of clinical outcome (pain and function) at three-, six-, and 12-months post MSK-triage appointment. 

Secondary aims are to: 

1. Measure self-reported use of healthcare resources over the 12-month follow-up period post MSK-triage appointment.  

2. Explore MSK phenotypes based on common prognostic factors for MSK pain.

## Methods

### Study design

ADAPT MSK is a prospective, observational, cohort study. The STROBE standardised reporting guidelines will be used to guide the reporting of this study ^22^. Adults with MSK pain attending CSP-led MSK triage clinics will be recruited from five sites across Ireland. Baseline assessment will consist of baseline demographics, work-related factors, healthcare utilisation and self-report questionnaires. Follow-up at 3, 6 and 12 months will involve repeat measurement of work-related factors, healthcare utilisation and self-report questionnaires.

### Ethics

Ethical approval for this study was granted by the Research Ethics Committees in Beaumont Hospital (Ref: 22/34), Tallaght University Hospital (Ref: 2418), Merlin Park Hospital (Ref: C.A. 2870), Midlands Regional Hospital Tullamore (Ref: RRECB1022FC) and St Vincent’s University Hospital (Ref: RS23-010). Written informed consent will be obtained from eligible participants prior to study recruitment, in line with the Data Protection Act 2018 (Section 36(2)) ^23^.

### Setting

This study will be based in MSK Triage clinics across five urban and regional secondary care sites in Ireland. These clinics are run by CSPs with more than five years clinical experience and the majority achieving a postgraduate MSc or PhD degree, in the field of MSK physiotherapy ^5^. They provide expert assessment, diagnosis and education to patients and identify the most appropriate management pathway for patients with MSK disorders. Patients on orthopaedic and rheumatology waiting lists, deemed unlikely to require orthopaedic surgeon or rheumatology consultant care are triaged to these MSK triage clinics, which improves service efficiency by reducing secondary care waiting lists and directing patients towards the appropriate care pathway ^7^.

### Participants

A consecutive sample of patients presenting to orthopaedic and rheumatology MSK-triage clinics with pain will be recruited. Participants will be eligible if they are aged 18 years or over, are triaged for non-consultant care at one of the five participating MSK triage services across Ireland, and have sufficient English language proficiency for the completion of self-reported questionnaires.

Patients will be ineligible to participate if they’ve been triaged by the CSP for orthopaedic surgical or rheumatologist assessment, are unable to communicate in English (written and spoken word), along with those who present with clinical indicators of suspected ‘red flag’ pathology (e.g. recent trauma with significant injury; acute, red, hot, or swollen joints; suspected fracture; joint infection; cancer) ^24^; or a diagnosed systemic inflammatory MSK condition (such as rheumatoid arthritis) or a diagnosis of dementia or terminal illness.

### Sample size

The estimated sample size is based on our primary aim. Approximately 18 predictor variables will be included in univariate analysis and with 10 events required per predictor variable ^25^, a sample of 180 participants is required. An additional 40% has been added to allow for drop-out at the 12-month follow-up, resulting in a final sample size of 252. 

### Recruitment and data collection

The MSK triage physiotherapist will identify and screen prospective participants for eligibility. If eligible, they will provide a participant information leaflet, briefly explain the objective of the study, and obtain written consent to be contacted by the primary investigator (FC). This allows the primary investigator to contact prospective participants to answer any questions about the study and if interested in participating, obtain informed written or electronic consent.

Once recruited, each participant will undergo a baseline assessment with the primary investigator, capturing participant demographics and healthcare utilisation, via Microsoft Teams or telephone, depending on participant preference. Thereafter, participants will complete a number of self-report questionnaires based on established prognostic factors i.e., baseline function, pain intensity, mental wellbeing, symptom duration, fear avoidance/catastrophising, quality of life/self-efficacy, widespread pain, age, co-morbidities, work absence duration, and education level ^15, 26, 27^. This data will be collected through Research Electronic Data capture (REDCap) software ^28, 29^, hosted at RCSI, on their personal device, or via posted paper questionnaires.

Demographic information will include participant gender, age, level of education, presenting MSK complaint, duration of symptoms, number of MSK pain sites, previous physiotherapy/surgery for presenting complaint, and work-related factors (employment status, work classification and duration of any work absence). Co-morbidities will be identified from a list of 12 comorbid conditions, informed by the National Institute of Clinical Excellence (NICE) indicator for multi-morbidity in primary care ^30^. Health literacy will be explored using the single-item literacy screener ^31^. Healthcare utilisation will be recorded using a modified version of the Managing of OSteoArthritis In ConsultationS (MOSAICS) trial questionnaire ^32^, which captures advice and information received about their condition, self-management, prescribed medications, aids and appliances, private/public health services (e.g., physiotherapy, GP, nursing, occupational therapy, podiatry), treatments, and investigations.

Self-report questionnaires will include the Musculoskeletal Health Questionnaire (MSK-HQ) ^33^, STarT MSK tool ^17^, and Patient Specific Functional Scale (PSFS) ^34^ to assess functional and MSK health status; pain intensity through the Numerical Pain Rating Scale (NPRS) ^35, 36^; fear of movement through the 11-item Tampa Scale for Kinesiophobia ^37^ and psychological distress via the Hospital Anxiety and Depression scale (HADS) ^38^.

All participants recruited in two sites (Beaumont Hospital and Tallaght University Hospital) will be invited to participate in a once-off baseline physical examination, consisting of grip strength examination, neurological exam, and quantitative sensory testing ( Table 1).

**Table 1.** Overview of primary and secondary outcomes, predictor variables, and time of assessment.

Variables		Method	Baseline	3 months	6 months	12 months
Primary Outcomes						
Pain Function	Numerical pain rating scale Mean of: - Current - Worst in last 24 hours - Least in last 24 hours Patient specific functional scale (PSFS)	RC RC	✓	✓ ✓	✓ ✓	✓ ✓
Secondary Outcomes						
Employment	Work status Work absence Work absence duration	MT/T	✓ ✓ ✓	✓ ✓ ✓	✓ ✓ ✓	✓ ✓ ✓
MSK Health Status	Musculoskeletal Health Questionnaire (MSK-HQ) STarT MSK	RC	✓	✓	✓	✓
Healthcare Utilisation	Modified MOSAICS Questionnaire	MT/T	✓	✓	✓	✓
Predictor Variables						
Demographics	Age Gender Education	MT/T	✓ ✓ ✓			
Baseline Clinical Factors	Total number of MSK pain sites (number/11 on body chart) NICE multi-morbidity index Single item health literacy screener (SILS) Duration of symptoms Previous surgery Previous physiotherapy	MT/T	✓ ✓ ✓ ✓ ✓ ✓	✓	✓	✓
Employment	Work classification	MT/T	✓	✓	✓	✓
Fear of Movement	Tampa Scale for Kinesiophobia (TSK-11)	RC	✓			
Anxiety and Depression	Hospital anxiety and depression scale (HADS)	RC	✓			
Optional Physical Examination (Two Recruitment Sites)						
Pain Phenotype	Quantitative Sensory Testing - Pressure pain threshold - Dynamic mechanical allodynia - Heat pain threshold - Temporal summation Clinical Neurological Exam	Physical	✓ ✓			
Grip Strength	Hand-held dynamometer	Physical	✓			

MSK, Musculoskeletal; MT/T, Microsoft Teams/Telephone; RC, RedCap

**Figure 1.**
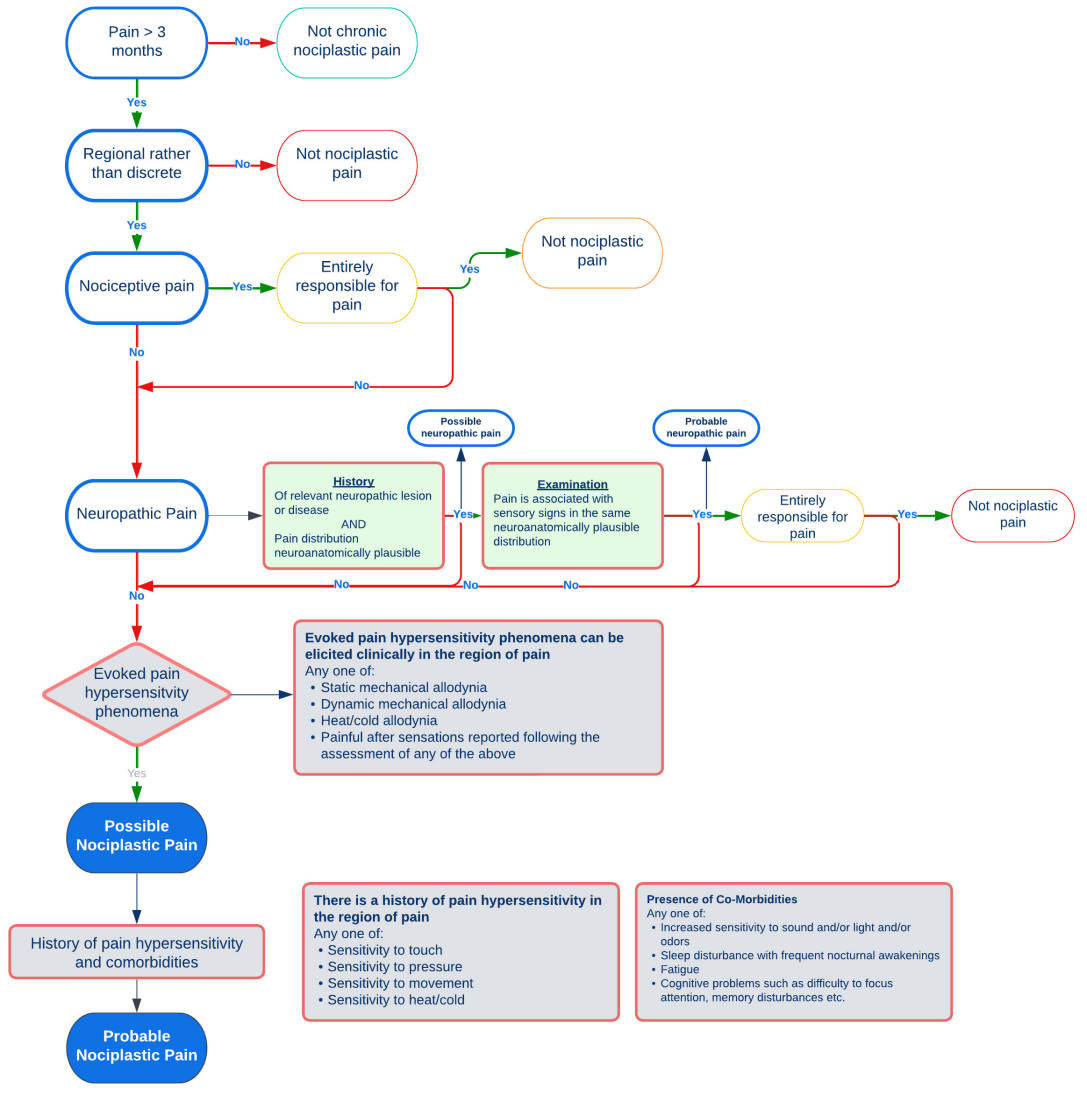
Screening process for pain classification based on IASP criteria for nociplastic pain ^41^ and NeuPSIG grading system for Neuropathic pain ^42^.

Pain hypersensitivity, measured by quantitative sensory testing, has been shown to be a predictor of worse outcome (pain and disability) at follow-up across multiple MSK conditions (e.g., osteoarthritis, low back pain, whiplash, post-operative pain) and different body sites (e.g., hip, knee, low back, shoulder and neck) ^13^. Quantitative sensory testing uses standardised testing protocols of somatosensory nerve function, to investigate potential underlying pain mechanisms ^39, 40^. The International Association for the Study of Pain (IASP) task force clinical criteria and grading system for nociplastic pain involves a stepwise approach to differentiate between predominant nociceptive, neuropathic or nociplastic pain ^41^, which, in conjunction with the NeuPSIG guidelines on neuropathic assessment ^42^ will be used to categorise participants’ dominant pain phenotype ( Figure 1). A quantitative sensory testing protocol including pressure pain thresholds (PPT), dynamic mechanical allodynia, pinprick, temporal summation and cold pain thresholds will be used to assess pain sensitivity in accordance with IASP and NeuPSIG grading systems ^41, 42^.

Grip strength is regarded as a biomarker of current health status and has been adopted as a singular indicator of overall body strength ^43– 45^. Grip strength will be assessed isometrically using a calibrated Jamar Plus Digital dynamometer following a standard protocol ^46^.

### Follow-up assessment

The primary investigator will contact participants at three, six, and 12 months via Microsoft Teams or telephone to collect healthcare utilisation data and work-related factors (employment status, work classification and duration of any work absence). Self-report questionnaires (MSK-HQ, STarT MSK, Patient Specific Functional scale, and NPRS) will be sent electronically via REDCap software or via post. Any participant withdrawals or loss to follow-up will be recorded.

### Outcomes

The primary outcomes of interest are pain intensity (NPRS) and function (PSFS). Secondary outcomes are musculoskeletal risk stratification status (STarT MSK), musculoskeletal health (MSK-HQ), healthcare utilisation and work-related factors (employment status, work classification, work absence ± duration).

### Statistical analysis

Pseudonymised data will be stored in an encrypted and password protected folder on the study’s SharePoint site in RCSI. Secure and encrypted access to the Microsoft SharePoint folder will be assigned to data controllers only. Descriptive statistics will be used to profile the characteristics of the cohort at baseline, three, six, and 12 months. Changes at three, six, and 12 months will be analysed using repeated measures multivariable regression. All models will be adjusted for potential confounding factors, checking for interactions and collinearity. Multivariable linear regression will be used to identify baseline predictors of pain and function outcomes at three, six, and the primary timepoint of 12 months. Variables included in the multivariable regression model will be selected if deemed clinically significant, or, if they have a univariable p-value of <0.2. Latent Class Analysis will be undertaken to explore underlying pain phenotypes within the cohort at baseline, three, six, and 12 months based on a range of observed categorical variables. Statistical significance will be inferred when the P value is <0.05. STATA 17 statistical software (StataCorp, College Station, Tx, USA) will be used for statistical analyses.

## Dissemination

Findings from this study will be disseminated via peer-reviewed journal publication and presentation at national and international conferences. Engagement with a public patient involvement (PPI) panel will explore dissemination strategies for public and service user engagement.

## Study status

Data collection commenced in December 2022, with study completion anticipated in November 2024.

## Discussion

The burden of MSK disorders is increasing exponentially worldwide, resulting in significant pressure on healthcare systems. People with MSK pain who present to their GP in Ireland are faced with difficulties accessing first-line public services, such as primary care physiotherapy and subsequently specialised orthopaedic and rheumatology services. To address secondary care waiting lists and improve service efficiency, the National MSK Triage Initiative, MSK triage clinics, run by CSPs under the clinical governance of Orthopaedic and Rheumatology Consultants commenced in Ireland in 2011, and has demonstrated success as a waiting list initiative. However, high discharge rates and onward referral to primary care physiotherapy following MSK triage suggest that these patients may have been managed more appropriately in primary care if sufficiently resourced. Currently, the patient journey and long-term outcomes following their MSK triage attendance are unknown. This longitudinal cohort study aims to identify predictors of pain and function outcomes up to 1 year following MSK triage attendance; measure individuals’ self-reported use of healthcare resources and explore MSK phenotypes based on identified prognostic factors. This research has the potential to inform future needs within primary care for those with MSK conditions, as well as the implementation of pathways from primary to secondary care orthopaedics and rheumatology, ensuring that patients receive the ‘right care, at the right place, at the right time’ in line with SláinteCare principles ^11^.

## Data Availability

No data are associated with this article.
