# Supplementary material for: ADvAnced PhysioTherapy in MuSculosKeletal Triage: Investigating prognostic factors, healthcare utilisation and clinical outcomes (ADAPT MSK) - a cohort study protocol
Source: HRB Open Res. 2025 Jan 23;6:73. Originally published 2023 Dec 6. [Version 2] doi: 10.12688/hrbopenres.13769.2 (PMC10879754; doi:10.12688/hrbopenres.13769.2)
Supplement: Peer review history

# Reviewer report: Reviewer response for version 2

**DOI:** 10.21956/hrbopenres.15444.r45241

**Published:** 2025-02-25

**Authors:** Shahnawaz Anwer

**Affiliations:** 1 The Hong Kong Polytechnic University, Hong Kong, Hong Kong

The protocol of this cohort study primarily aimed to identify predictors of clinical outcome (pain and function) at 12-months post MSK-triage appointment. Secondarily, this study aimed to describe the clinical course of patients attending MSK triage clinics and measure self-reported use of healthcare resources up to 12 months post-MSK-triage appointment. The topic is interesting and useful. The study design is well planned and feasible. However, there are some inconsistencies in reporting primary and secondary outcomes. 

 Comments:

 1. There are inconsistencies in pain measurements. For example, if pain intensity is assessed through multiple methods, it should be consistently defined and reported to avoid ambiguity. 

 2. Some outcomes are not included in all follow-up assessments. For example, if certain outcomes such as health literacy, Fear of Movement, Anxiety and Depression, and grip strength are recorded at baseline but not during follow-ups, it may leave gaps in the understanding of how these factors evolve over time.

 3. Protocol should identify and report any potential limitations and how to address those limitations.

Is the study design appropriate for the research question?

Yes

Is the rationale for, and objectives of, the study clearly described?

Yes

Are sufficient details of the methods provided to allow replication by others?

Yes

Are the datasets clearly presented in a useable and accessible format?

Not applicable

Reviewer Expertise:

Rehabilitation, Osteoarthritis, Musculoskeletal Research, Ergonomics, Physical Fatigue

I confirm that I have read this submission and believe that I have an appropriate level of expertise to confirm that it is of an acceptable scientific standard, however I have significant reservations, as outlined above.

# Reviewer report: Reviewer response for version 1

**DOI:** 10.21956/hrbopenres.15061.r37418

**Published:** 2024-02-20

**Authors:** helen o'leary

**Affiliations:** 1 University of Limerick, Limerick, County Limerick, Ireland

**Introduction**

 Overall could include more information to expand on the gap this research is addressing/what is known already and why this research is important

 While the background contains interesting info on the MSK triage initiative it’s not clear from the Introduction why this is an important question? Why is knowing predictors of outcome for this particular patient cohort at 1 year is important? What are the implications of better predicting outcomes? Could it be used for early identification of those at risk of persistent pain and subsequent prioritisation by physiotherapy services? How will this information be used to improve or better current MSK services in Ireland? (some mention of this in Discussion)

 Also, while the authors refer to previous SRs of predictors of pain and function in MSK conditions, it would be useful to know what type of patient population these relate to (primary care or secondary care). It is stated that MSK triage clinics have not been studied, which is true, however this cohort are essentially very similar to cohort attending secondary care elective othopaedic clinics, what are the main findings from these studies?

**Methods**

 Study Design- Follow-up at 3, 6 and 12 months- is there a pre-specified primary end-point?

 How many are estimated to undergo a baseline physical exam? Will there be sufficient data from these two sites to carry out the anticipated data analysis e.g. latent class analysis or multi-variable analysis? Or is this info solely to categorise the pain phenotype? Should make reference to this sub-group in the data analysis section.

 Pain phenotyping-  using IASP criteria and NeuPSIG based on baseline physical exam. Is this different to the Latent Class Analysis to explore underlying pain phenotypes “based on a range of categorical variables”? Clarify this.

 How soon will the physical exam be after the patient is recruited? What is the anticipated duration of this examination  eg. 1 hour?

 QST- Would have expected in a protocol to see more detail here in order that your testing procedures are reproducible. For example how do you intend to choose anatomical testing sites (related to the primary pain complaint?) What is the order of testing? Instruments- Device you are using for pressure pain algometry, what thermal testing devices?. What is pinprick testing? References (41, 42) provided are for grading systems and not for a QST protocol. Did the authors mean to reference the Rolke et al, 2006 (40)DFNS protocol here? 

 If DFNS Rolke 2006 protocol is what the researchers are using, are the researchers following a modified version of DFNS Rolke 2006 protocol or reproducing exactly. For example, this protocol uses testing of 1 pinprick versus 10 repeated pinpricks and repeats this procedure 5 times, this (55 pinpricks !) may be poorly tolerated by patients, esp hyperalgesic. Have the researcher’s modified this? How?

 Perhaps Table 1 could include more detail on protocol or how differs to referenced protocol or include as supplementary info

 Table 1- different QST info compared to text- pinprick omitted, heat pain threshold here instead of cold pain thresholds

 Clinical Neurological Exam- indicate in Table 1 what will this entail? Is the main purpose to help with pain classification?

  Patients attending Rheumatology MSK triage clinics are typically multi-site MSK pain, eg. Small joints of the hands plus other several painful area. It is not clear how it's decided which is the index site for the primary outcome pain NRS. Similarly for QST indicate how will select test site?

Is the study design appropriate for the research question?

Yes

Is the rationale for, and objectives of, the study clearly described?

Partly

Are sufficient details of the methods provided to allow replication by others?

Partly

Are the datasets clearly presented in a useable and accessible format?

Not applicable

Reviewer Expertise:

musculoskeletal pain, knee pain

I confirm that I have read this submission and believe that I have an appropriate level of expertise to confirm that it is of an acceptable scientific standard.

# Response

**Published:** 2025-01-20

**Authors:** Fiona Callan

**Affiliations:** Physiotherapy, RCSI, Dublin, Dublin, Ireland

Thank you, Dr O’Leary, for your time and expertise in reviewing our protocol. We greatly appreciate all your comments and suggested amendments. We have reviewed and addressed each point you have raised below, and amended the paper accordingly.

**Reviewer comment 1:**

**While the background contains interesting info on the MSK triage initiative it’s not clear from the Introduction why this is an important question? **

1. **Why is knowing predictors of outcome for this particular patient cohort at 1 year is important?**
2. **What are the implications of better predicting outcomes? Could it be used for early identification of those at risk of persistent pain and subsequent prioritisation by physiotherapy services? **
3. **How will this information be used to improve or better current MSK services in Ireland? (some mention of this in Discussion)**

Whilst we know from the pre-existing literature that MSK triage is effective at decreasing waiting times for secondary care orthopaedics and rheumatology (Fennelly et al., 2018), we do not know what happens to these patients following MSK triage attendance. There is no national research on patient outcomes or what factors may predict patient outcomes in this cohort. The high discharge rate from MSK triage warrants further exploration. It is potentially a consequence of insufficient resources in primary care services resulting in referring practitioners generating referrals to multiple services in primary and secondary care to access the quickest care, and not necessarily the right care at the right time, in line with the Irish government health reform plan (Burke et al., 2018). Identifying predictors of outcome (pain and function) at 12 months has the potential to inform decision making on the optimal patient pathway and trajectory of care by enabling the identification of those at risk of a poor outcome at 1 year, earlier in their journey.

 Burke S, Barry S, Siersbaek R, Johnston B, Ní Fhallúin M, Thomas S. Sláintecare - A ten-year plan to achieve universal healthcare in Ireland. Health Policy. 2018 Dec;122(12):1278-1282. doi: 10.1016/j.healthpol.2018.05.006. Epub 2018 May 22. PMID: 29843901.

 Fennelly O, Blake C, FitzGerald O, Breen R, Ashton J, Brennan A, Caffrey A, Desmeules F, Cunningham C. Advanced practice physiotherapy-led triage in Irish orthopaedic and rheumatology services: national data audit. BMC Musculoskelet Disord. 2018 Jun 1;19(1):181. doi: 10.1186/s12891-018-2106-7. PMID: 29859072; PMCID: PMC5984783.

**Reviewer comment 2:**

**Also, while the authors refer to previous SRs of predictors of pain and function in MSK conditions. It would be useful to know what type of patient population these relate to (primary care or secondary care).**

 The manuscript has been updated to reflect this in the introduction section.

**Reviewer comment 3:**

**It is stated that MSK triage clinics have not been studied, which is true, however this cohort are essentially very similar to cohort attending secondary care elective orthopaedic clinics, what are the main findings from these studies?**

 As the focus of this study is on patients who do not receive specialist orthopaedic or rheumatology care, rather than all patients who attend elective orthopaedic clinics, the below sentence in the introduction has been reworded as follows for clarification. Additionally, we’re primarily interested in participants clinical course (i.e. healthcare utilisation) and outcomes over 1 year **following** their MSK triage appointment. The findings from our study will be discussed in comparison with pre-existing literature in the published paper.

 ‘H *owever, currently, patient outcomes, prognostic stratification, and predictors of outcome up to 1-year later have not been consistently studied in patients attending MSK triage clinics* *, who do not require referral to specialist orthopaedic or rheumatology care,* *in Ireland or internationally’.*

# Reviewer report: Reviewer response for version 1

**DOI:** 10.21956/hrbopenres.15061.r37424

**Published:** 2024-02-01

**Authors:** Karin Samsson

**Affiliations:** 1 University of Gothenburg, Gothenburg, Sweden

This is an important aspect to address in research, to improve on the knowledge regarding the patients that are referred for MSK triage. Furthermore, to be able to screen patients prior to referral could reduce waiting times as well as improve care on the right level. 

1. You state in the introduction that 71% of patients were discharged at their initial appointment and 23% referred to physiotherapy, which makes me wonder. The 71% who were discharged, do you think that they would have needed different care than what they received? And since this large number of patients were discharged, and not referred to physiotherapy, why do you think predictors of clinical outcome and prognostic factors for MSK pain. is important to investigate for this patients group?
2. The aims are not consistently reported throughout the paper. 
3. You have a great number of predictors and outcomes, but I struggle to make them out clearly since they are inconsistently reported in introduction and various sections in the methods. It would be highly beneficial for the reader to have these clearly and consistently presented regarding what outcome you want to measure and what outcome measure you use. Furthermore, to use the same terms i.e. in some sections you use workers' sick leave, sick leave or work-related factors. 
  Participants​​​
4. Regarding patients included in the study - who is responsible for deciding which patients are "unlikely to require consultant care" and based on what? 
5. Are the patients to be included in the study after the triage? When is the patient asked to participate? And why don’t you include the patients in need of orthopaedic surgeon to be able to investigate predictors of clinical outcome and prognostic factors for MSK pain for them as well? Considering that 71% are discharged, it would probably be quite beneficial to know if there is a difference between these two groups.
  Sample size
6. There is no formal sample size calculation.
  Recruitment and data collection
7. You state that the MSK triage physiotherapist will identify and screen prospective participants for eligibility – is this before the actual triage? I think this process needs to be clearly described (as also addressed in previous question).
8. If I understand correctly you are collecting two written consents? What is the purpose of that?
9. Why did you chose to have a teams or telephone data collection as well as questionnaires?
10. Inconsistent reporting of outcomes collected and through what outcome measures, order and the use of abbreviations or not.  Furthermore not clear which ones are collected via self-reported questionnaires vs telephone/teams. Furthermore, not all outcomes are included in the primary or secondary outcomes.
11. The outcome health literacy is not mentioned or described prior to data collection.
12. The whole section starting with “Pain hypersensitivity, measured by… / (Figure 1) seems more to belong in the introduction section. 
  Outcomes
13. Outcomes are not consistent with aims. 

Is the study design appropriate for the research question?

Yes

Is the rationale for, and objectives of, the study clearly described?

Partly

Are sufficient details of the methods provided to allow replication by others?

Partly

Are the datasets clearly presented in a useable and accessible format?

Not applicable

Reviewer Expertise:

Physiotherapist led orthopaedic triage. Have not focused on the statistics part as that is not my area of expertise.

I confirm that I have read this submission and believe that I have an appropriate level of expertise to confirm that it is of an acceptable scientific standard, however I have significant reservations, as outlined above.

# Response

**Published:** 2025-01-20

**Authors:** Fiona Callan

**Affiliations:** Physiotherapy, RCSI, Dublin, Dublin, Ireland

Thank you, Dr Samsson, for your time and expertise in reviewing our protocol. We greatly appreciate all your comments and suggested amendments. We have reviewed and addressed each point you have raised below, and amended the paper accordingly.

**Reviewer Comment 1:**

**You state in the introduction that 71% of patients were discharged at their initial appointment and 23% referred to physiotherapy, which makes me wonder. The 71% who were discharged, do you think that they would have needed different care than what they received? And since this large number of patients were discharged, and not referred to physiotherapy, why do you think predictors of clinical outcome and prognostic factors for MSK pain is important to investigate for this patients group?**

 Currently, we do not know from the literature why there is a high discharge rate, nor why a lower percentage are referred to physiotherapy. Historically waiting times to access primary care physiotherapy and secondary care services in Ireland (e.g. pain management, orthopaedics, rheumatology) are long, resulting in delays to patients accessing MSK care. It is hypothesised that many patients are referred for imaging, primary care physiotherapy and secondary care orthopaedics or rheumatology simultaneously in order to receive the quickest care in the absence of a resourced stepwise integrated care pathway. Therefore, we wanted to study the cohort of patients who attended secondary care MSK triage services and did not require surgical or medical input from the orthopaedic surgeon or rheumatologist to better understand their healthcare journey (healthcare utilisation), clinical outcomes (pain and function) and predictors of outcome. We have reflected the importance of this research study in the introduction section.

**Reviewer Comment 2:**

**The aims are not consistently reported throughout the paper.**

 We have updated and addressed the aims of our study in the introduction, methods and discussion sections.

**Reviewer comment 3:**

**You have a great number of predictors and outcomes, but I struggle to make them out clearly since they are inconsistently reported in introduction and various sections in the methods. It would be highly beneficial for the reader to have these clearly and consistently presented regarding what outcome you want to measure and what outcome measure you use. Furthermore, to use the same terms i.e. in some sections you use workers' sick leave, sick leave or work-related factors.**

 Primary outcomes, secondary outcomes and predictor variables are outlined in Table 1, along with their method of collection (e.g. Microsoft teams/telephone or REDCap/postal questionnaire).

**Reviewer comment 4:**

**Regarding patients included in the study - who is responsible for deciding which patients are "unlikely to require consultant care" and based on what?**

 Participants in this study will be recruited after attending a MSK triage clinic in a participating site. There, a MSK triage physiotherapist will triage the patient and decide if the patient requires consultant review for surgical or medical management, or alternative management options (e.g. discharge to GP, physiotherapy referral, referral for imaging, pain medicine referral etc.) as is their normal clinical practice. Only those patients who do not require consultant referral following MSK triage review are eligible for inclusion in this study. It’s at this point, once the decision for non-consultant care is decided and eligibility is determined, that participants are invited to participate in the study by the MSK triage physiotherapist.

**Reviewer comment 5:**

**Are the patients to be included in the study after the triage? When is the patient asked to participate? And why don’t you include the patients in need of orthopaedic surgeon to be able to investigate predictors of clinical outcome and prognostic factors for MSK pain for them as well? Considering that 71% are discharged, it would probably be quite beneficial to know if there is a difference between these two groups.**

 The purpose of this research was to evaluate the patient journey through MSK triage services in Ireland of those who, in reality may never have needed to attend secondary care. Whilst lots of research has reported the percentage of patients who are discharged from MSK triage, without needing specialist referral, there is limited research on their outcomes up to one–year. Therefore, we excluded those patients who required onward referral to consultant orthopaedic surgeons or rheumatologists, as they may often require surgical management (e.g. joint replacement surgery) or medical management of a systemic condition (e.g. inflammatory arthritis). There is also a cohort of patients, whom following GP referral, are seen directly by the consultant, following triaging of referrals. This cohort of patients do not attend MSK triage and are instead managed directly by the consultant. Therefore, we wanted to focus on the cohort of patients who did not require consultant led surgical or medical management to evaluate their outcomes (pain and function) and healthcare utilisation following MSK triage and identify predictors of outcome, which may help inform the development of modernised care pathways for the management of musculoskeletal pain.

**Reviewer comment 6:**

**You state that the MSK triage physiotherapist will identify and screen prospective participants for eligibility – is this before the actual triage? I think this process needs to be clearly described (as also addressed in previous question).**

 This has been updated in the ‘Recruitment and Data Collection’ section to say “The MSK triage physiotherapist will identify and screen prospective participants for eligibility following their MSK triage appointment”. Therefore, eligibility can only be determined once the triage appointment is complete.

**Reviewer comment 7:**

**There is no formal sample size calculation.**

 Patient outcomes and potential explanatory variables associated with outcomes up to 1-year after musculoskeletal triage review have not been consistently studied in patients attending MSK triage clinics in Ireland or internationally, and thus there is limited information on potential explanatory variables associated with pain. We agree the number "10" is somewhat arbitrary, and its applicability can vary depending on the context and characteristics of the data (Vittinghoff and Mcculloch, 2007, Peduzzi et al., 1996). We used this as a guideline to the minimum sample size required, acknowledging practical constraints, and carefully considering explanatory variables to explore in this study based on theory and previous research.

 Please also refer to our response to Reviewer 1 regarding the sample size query

 Vittinghoff E, McCulloch CE. Relaxing the rule of ten events per variable in logistic and Cox regression. Am J Epidemiol. 2007;165(6):710-8.

 Peduzzi P, Concato J, Kemper E, Holford TR, Feinstein AR. A simulation study of the number of events per variable in logistic regression analysis. J Clin Epidemiol. 1996;49(12):1373-9.

**Reviewer comment 8:**

**If I understand correctly you are collecting two written consents? What is the purpose of that?**

 A cooling off period is required between the time of introduction to the study and the obtaining of informed consent. The researcher (FC) is not present at the MSK triage clinics and due to the busy nature of these clinics, it would not be feasible to allow for a cooling off period on the day. Therefore, MSK triage physios, upon introducing the study to the patient obtain consent to contact(i.e. be contacted by the research team) from interested patients. This allows the researcher (FC) to contact the participant the following day (12-24 hours later) to obtain informed consent and recruit participants to the study. This is in line with our research ethics requirements.

**Reviewer comment 9:**

**Why did you chose to have a teams or telephone data collection as well as questionnaires?**

 The patient demographic attending MSK triage clinics have varied information technology (IT) skills, therefore we decided it would be best to offer those with limited IT skills the ability to still participate by allowing for telephone call assessments and paper questionnaires.

 This sentence in the recruitment and data collection section has been updated as follows:

*‘This data will be collected through Research Electronic Data capture (REDCap) software (*28 *, *29 *), hosted at RCSI, on their personal device, or via posted paper questionnaires* *to facilitate participants with limited information technology skills* *.’*

**Reviewer comment 10:**

**Inconsistent reporting of outcomes collected and through what outcome measures, order and the use of abbreviations or not.  Furthermore not clear which ones are collected via self-reported questionnaires vs telephone/teams. Furthermore, not all outcomes are included in the primary or secondary outcomes.**

 All predictor variables and outcomes are outlined in table 1, along with their method of collection (e.g. Microsoft teams/telephone or REDCap/postal questionnaire). Consistency in reporting of outcomes/variables and the measures used has been revised in the new manuscript.

**Reviewer comment 11:**

**The outcome health literacy is not mentioned or described prior to data collection.**

 Health literacy is now included in the introduction and data collection sections along with table 1.

**Reviewer comment 12:**

**The whole section starting with “Pain hypersensitivity, measured by… / (Figure 1) seems more to belong in the introduction section.**

 This was included to further describe the quantitative sensory testing component of this project. Reference to altered pain processing is also included in the introduction section.

**Reviewer comment 13:**

1. **Outcomes are not consistent with aims. **

We have revised our aims to align with our outcomes in the revised manuscript.

# Reviewer report: Reviewer response for version 1

**DOI:** 10.21956/hrbopenres.15061.r37570

**Published:** 2024-01-16

**Authors:** Subhash Aryal

**Affiliations:** 1 University of Pennsylvania, Philadelphia, Pennsylvania, USA

I focused on the statistical analysis component of the manuscript. Here are my specific concerns regarding this manuscript.

 1) There is no formal sample size calculation. Rule of thumb of 10 events per variable is used. Software including PASS, SAS can actually provide a more formal sample size estimation.

 2) The authors propose using repeated measures multivariable regression. I recommend being more specific in terms of whether it is random-effects model, covariance pattern model or marginal GEE model.

 3) The authors have adjusted sample size to account for attrition. But missing data is not addressed in the statistical analysis plan.

 4) If the authors are going to run separate models for baseline predictors of pain and function outcomes at three, six and the primary timepoint of 12 months, they should adjust the statistical significance level to adjust for inflated type I error rate.

 5) There is no detail provided regarding the proposed Latent Class Analysis. 

 6) On page 4, column 2 the authors state "..... multiple MSK conditions  (e.g., osteoarthritis, low back pain, whiplash, post-operative pain). It is quite possible that a large majority with post-operative pain may be lost to follow-up at 12 months. If the participant's pain is not chronic, they may drop-out of the study.

Is the study design appropriate for the research question?

Yes

Is the rationale for, and objectives of, the study clearly described?

Yes

Are sufficient details of the methods provided to allow replication by others?

Partly

Are the datasets clearly presented in a useable and accessible format?

Not applicable

Reviewer Expertise:

Biostatist

I confirm that I have read this submission and believe that I have an appropriate level of expertise to confirm that it is of an acceptable scientific standard, however I have significant reservations, as outlined above.
